# *OsSAP6* Positively Regulates Soda Saline–Alkaline Stress Tolerance in Rice

**DOI:** 10.1186/s12284-022-00616-x

**Published:** 2022-12-27

**Authors:** Fengjin Zhu, Kai Wang, Danni Li, Ziang Liu, Minghui Li, Zhenyu Wang, Xiufeng Li, Xingguo Lan, Qingjie Guan

**Affiliations:** 1 Key Laboratory of Saline-Alkali Vegetation Ecology Restoration, Ministry of Education, College of Life Sciences, Northeast Forestry University, Harbin, 150040 China; 2 Northeast Institute of Geography and Agroecology, Key Laboratory of Soybean Molecular Design Breeding, Chinese Academy of Sciences, Harbin, 150081 China; 3 College of Forestry, Northeast Forestry University, Harbin, 150040 China

**Keywords:** Rice, Saline–alkaline stress, Transcription factors, *OsSAP6*, *OsPK5*

## Abstract

**Background:**

Soil salinization is a worldwide environmental problem, especially in the arid and semiarid regions of northeastern China, which are heavily affected by soda saline–alkaline stress. At present, there is an urgent need to improve the soda saline–alkaline stress tolerance of rice.

**Results:**

Stress-associated proteins are involved in regulating the abiotic stresses in plants. There are 18 members of the rice *stress-associated protein (OsSAP*) gene family. In this study, the expression levels of *OsSAP6* in leaves and roots were upregulated with increasing NaHCO_3_ stress duration. OsSAP6 was located in nucleus and cytoplasm. The bud length and total root length of *OsSAP6* overexpression rice were significantly longer than those of Lj11 (*Oryza sativa longjing11*) during germination stage, and the survival rates, plant height and malondialdehyde content at the seedling stage showed tolerance growth of saline–alkaline stress. The expression of *OsCu/Zn-SOD*, *OsAPX2*, and *OsCAT1* in transgenic lines was increased significantly under SAE (soda saline–alkali soil eluent) stress. OsSAP6 interacts with OsPK5 according to yeast two-hybrid screening and luciferase complementation experiments. The expression of *OsPK5* increased under NaHCO_3_ and H_2_O_2_ stress, and the overexpression of *OsPK5* in rice improved soda saline–alkaline tolerance.

**Conclusion:**

Overexpression of *OsSAP6* in rice significantly enhanced saline–alkaline tolerance compared with the wild type. It is speculated that *OsSAP6* responds to soda salinity stress and interacts with *OsPK5* to positively regulate soda saline–alkaline tolerance through ROS homeostasis. This study revealed the features of *OsSAP6* involved in response to soda saline–alkaline stress and the interaction with OsPK5, which provided resources for breeding aimed at improving the soda saline–alkaline stress tolerance of rice.

**Supplementary Information:**

The online version contains supplementary material available at 10.1186/s12284-022-00616-x.

## Background

According to a survey, saline–alkali land is distributed in 36 countries, with a total area of 956 million hectares (Yun and Chen 2020). Among those countries, the existing saline–alkali land in China is as high as 9.913 × 10^6^ hm^2^, mainly distributed in 23 provinces, including northeast, north, northwest and coastal areas. Those areas contain more than 10% of the total arable land in China with the potential for agricultural development. These lands are precious reserve arable land resources (Liu and Wang 2021; Zhu et al. 2018). The current research shows that rice cultivation is an effective method to control saline–alkali land. In the low-lying inland areas of the western Songnen Plain, the improvement measures for growing rice in saline–alkali land have achieved high economic benefits (Zhang and Wang 2016). Due to the expansion of rice cultivation areas and the requirement of high yield in the saline–alkali land of Northeast China, the rice varieties grown there should be more able to tolerate the saline–alkaline environment.

Stress-associated proteins (SAPs) are proteins containing A20/AN1 zinc finger domains with N-terminal A20 and/or C-terminal AN1 domains, and the expression of these genes changes when plants respond to stress (Evans et al. 2004; Huang et al. 2004). In recent years, studies have reported that many gene families and interaction networks are involved in regulating the response of rice to stress. There are 18 members of the rice *stress-associated protein (OsSAP*) gene family, which were determined based on a nucleotide alignment search of 1 kb of the A20/AN1 zinc finger domain, and quantitative expression analysis showed that most of these genes were induced by abiotic stress (Vij and Tyagi 2006). Other plants have been found that *SAPs* involved in abiotic stress tolerance responses, such as *AtSAP5*, *AtSAP10*, and *AtSAP12* in *Arabidopsis thaliana* (Kang et al. 2011; Dixit and Dhankher 2011; Ströher et al. 2009), *AlSAP* in *Aeluropus littoralis* (Ben Saad et al. 2012a, b), *MtSAP1* in *Medicago truncatula* (Gimeno-Gilles et al. 2011), *ShSAP1* in *Saccharum officinarum* (Li et al. 2011), *ZmAN13* in *Zea mays* (Xuan et al. 2011) and *MusaSAP1* in banana (Sreedharan et al. 2012), and those are resistant to adversity. Overexpression of *OsSAP1* in transgenic rice results in altered expression of several endogenous genes, including those coding for transcription factors, membrane transporters, signaling components, and genes involved in metabolism, growth and development (Dansana et al. 2014). The expression of *OsSAP1* was induced under a variety of abiotic stresses, including cold, salt, dehydration, submersion, heavy metals, ABA (abscisic acid) and injury, and involved in the regulation of multiple abiotic stresses, including the ROS (reactive oxygen species) pathway (Mukhopadhyay et al. 2004). *OsSAP8*, *OsSAP9*/*ZFP177* and *OsSAP11* have also been reported to have the ability to enhance abiotic tolerance (Kanneganti and Gupta 2008; Huang et al. 2008; Giri et al. 2011). Overexpression of *OsSAP8* in both transgenic tobacco and rice conferred tolerance to salt, drought, and cold stress at the germination and seedling stage, as reflected by the percentage of germination and gain in fresh weight after stress recovery, the transgenic plants were tolerant to salt and drought during the anthesis stage without any yield penalty compared to unstressed transgenic plants (Kanneganti and Gupta 2008). *ZmAN11*, the homolog of *OsSAP8* in maize, increased expression under NaCl, cold and heat-shock stresses and decreased under drought stress (Xuan et al. 2015). OsSAP7 exhibits E3 ubiquitin ligase activity in vitro. Overexpression of *OsSAP7* in Arabidopsis was insensitive to ABA at germination stage and sensitive to water-deficit stress at an advanced stage compared to the wild type. OsSAP7 was also impaired in ABA and stress-responsive gene expression, and acts as a negative regulator by acting as an E3 ubiquitin ligase (Sharma et al. 2015). The wheat *stress-associated protein 5* (*TaSAP5*) ubiquitinated substrate is HSP90C (chloroplast heat shock protein 90), and it is involved in drought regulation in wheat and Arabidopsis as an E3 ubiquitin ligase for the degradation of DRIP (dehydration-responsive element binding protein2a interacting protein) and MBP-1 (c-myc binding protein) (Zhang et al. 2017, 2019). The ubiquitin–proteasome system is an important protein regulatory mechanism that affects many cellular processes in plant growth, including stress responses (Lyzenga and Stone 2012; Dreher and Callis 2007), hormonal signaling (Kelley and Estelle 2012), embryogenesis (Hellmann et al. 2003), and intracellular transport (Lee et al. 2009). Two mutants with overexpression of *OsSAP16* showed reduced stomatal conductance in rice leaves, but there were no differences in stomatal development or morphology in either of the mutants. This phenotype limited CO_2_ uptake and the rate of photosynthesis, resulting in less biomass accumulation in the two mutants. Whole transcriptome analysis showed that overexpression of *OsSAP16* led to global changes in gene expression. These results show that *OsSAP16* was involved in modulating the response of rice to drought stress by regulating the expression of a set of stress-associated genes (Wang et al. 2016).

Transcription factors played important regulatory roles in plant responses to different stresses (Singh et al. 2002). *OsSAPs* are involved in abiotic stress response pathways as transcriptional regulators and have different regulatory roles. Vij and Tyagi (2006) showed that *OsSAP4*, *OsSAP6*, *OsSAP8*, *OsSAP9*, *OsSAP15*, and *OsSAP16* seemed to have a high level of expression in the unstressed state, and the mRNA level of all the *OsSAPs* showed an increase in response to salt and dehydration stress treatment for 6 h relative to the unstressed control. We detected the expression of *OsSAP4*, *6*, *8*, and *9* in the same clade by quantitative real-time PCR (qRT–PCR) under 60 mM NaHCO_3_ stress and found that the expression of *OsSAP6* increased the most. To determine the regulatory function of *OsSAP6* in response to saline–alkaline stress, the *OsSAP6* gene was cloned. Subcellular location of OsSAP6 was verified in onion epidermal cells. The resistance to SAE (soda saline–alkali soil eluent) stress at germination and seedling stages was analyzed with overexpression of *OsSAP6* in Lj11 (*Oryza sativa longjing11*). The interacting protein OsPK5 was screened by yeast two-hybrid (Y2H) assay and then verified. The increased expression and enhanced tolerance to alkaline stress in rice overexpressing *OsPK5* were investigated. In this study, we investigated the function of *OsSAP6* regulating soda salinity resistance in rice, revealed the synergistic relationship between OsSAP6 and interacting protein in regulating salinity resistance, and provided genetic resources and new insights for soda saline–alkaline resistance breeding.

## Results

### Expressional Patterns of ***OsSAP4***, ***6***,*** 8***, and ***9*** Under NaHCO_3_ Stress

**Fig. 1:**
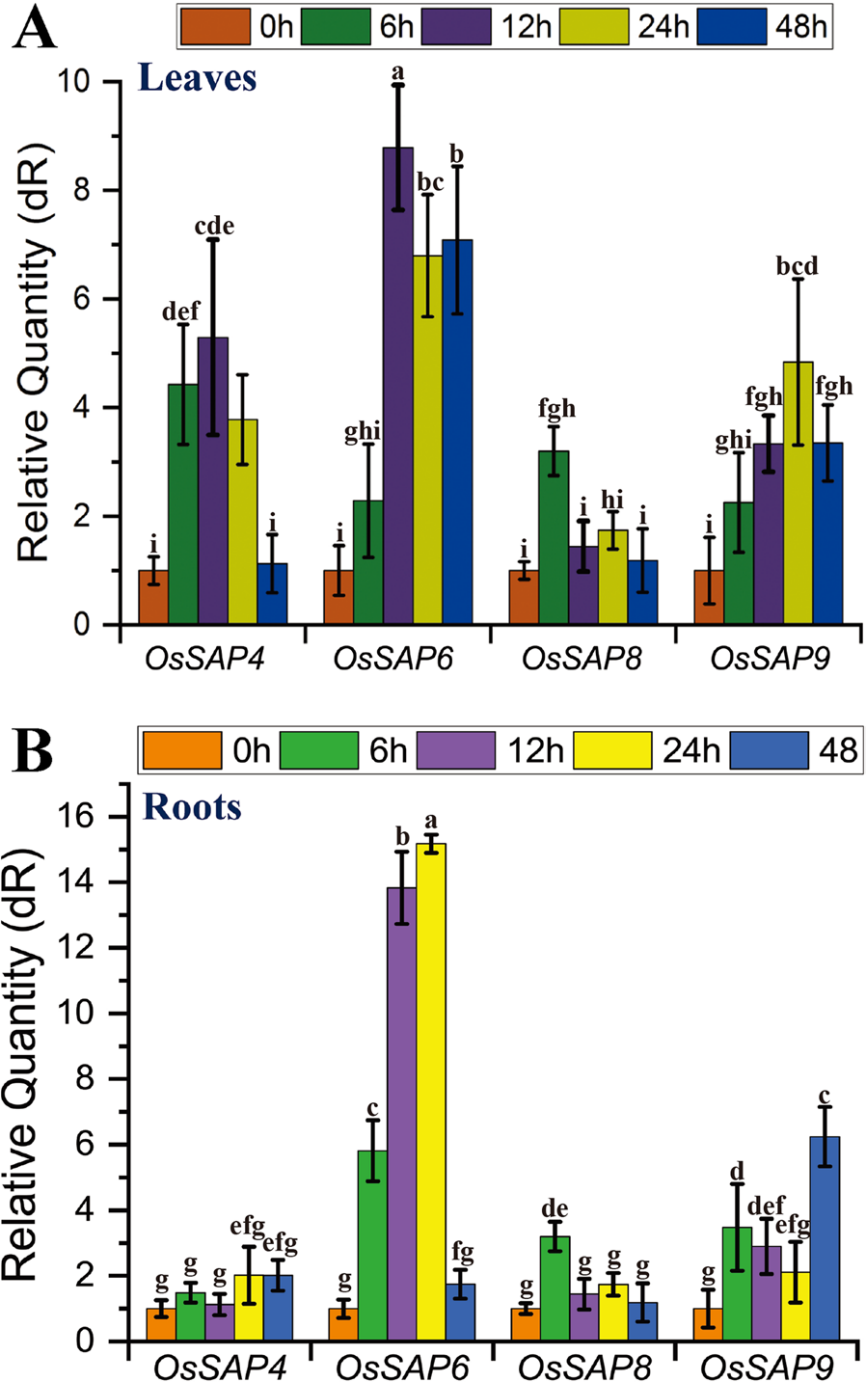
The expression of *OsSAP4*, *6*, *8*, and *9* under 60 mM NaHCO_3_. Lj11 seedlings at the three-leaf stage were treated with 60 mM NaHCO_3_ for 0, 6, 12, 24, and 48 h to detect the expression of *OsSAP4*, *6*, *8*, and *9* in leaves (**A**) and roots (**B**). The expression level of the four genes at 0 h was set to 1, and the *Os18sRNA* gene was used as an internal reference control. The fold change was analyzed by the 2^−ΔΔCT^ method. Values are the mean ± standard deviation of three biological replicates. Statistical differences are labeled with different letters using Duncan test (*p* < 0.05, one-way ANOVA)

Based on the A20 and AN1 domains of *OsSAPs*, 18 homologous genes were found in NCBI GenBank database (https://www.ncbi.nlm.nih.gov/), details are shown in Additional file 2: Table S1. Phylogenetic analysis of *OsSAPs* showed that *OsSAP4*, *6*, *8*, and *9* are placed in the same clade (Additional file 1: Fig. S1, S2). This group has been reported to be closely related to abiotic stress responses in plants. Further expressional analysis results showed that all these four genes were responsive to NaHCO_3_ stress (Fig. 1). *OsSAP6* was the most upregulated one, which was increased by 8.8 times compared to the control at 12 h. The increased expression of the four genes in the roots varied considerably. *OsSAP4*, *8*, and *9* were less increased than *OsSAP6*. The expression level of *OsSAP6* at 24 h was 15.2 times higher than that of the control. The strong response of the *OsSAP6* to NaHCO_3_ stress in roots suggests that the main biological function of this gene may be the regulation of NaHCO_3_ stress.

### Subcellular Localization and Organizational Expression of OsSAP6 Protein

**Fig. 2:**
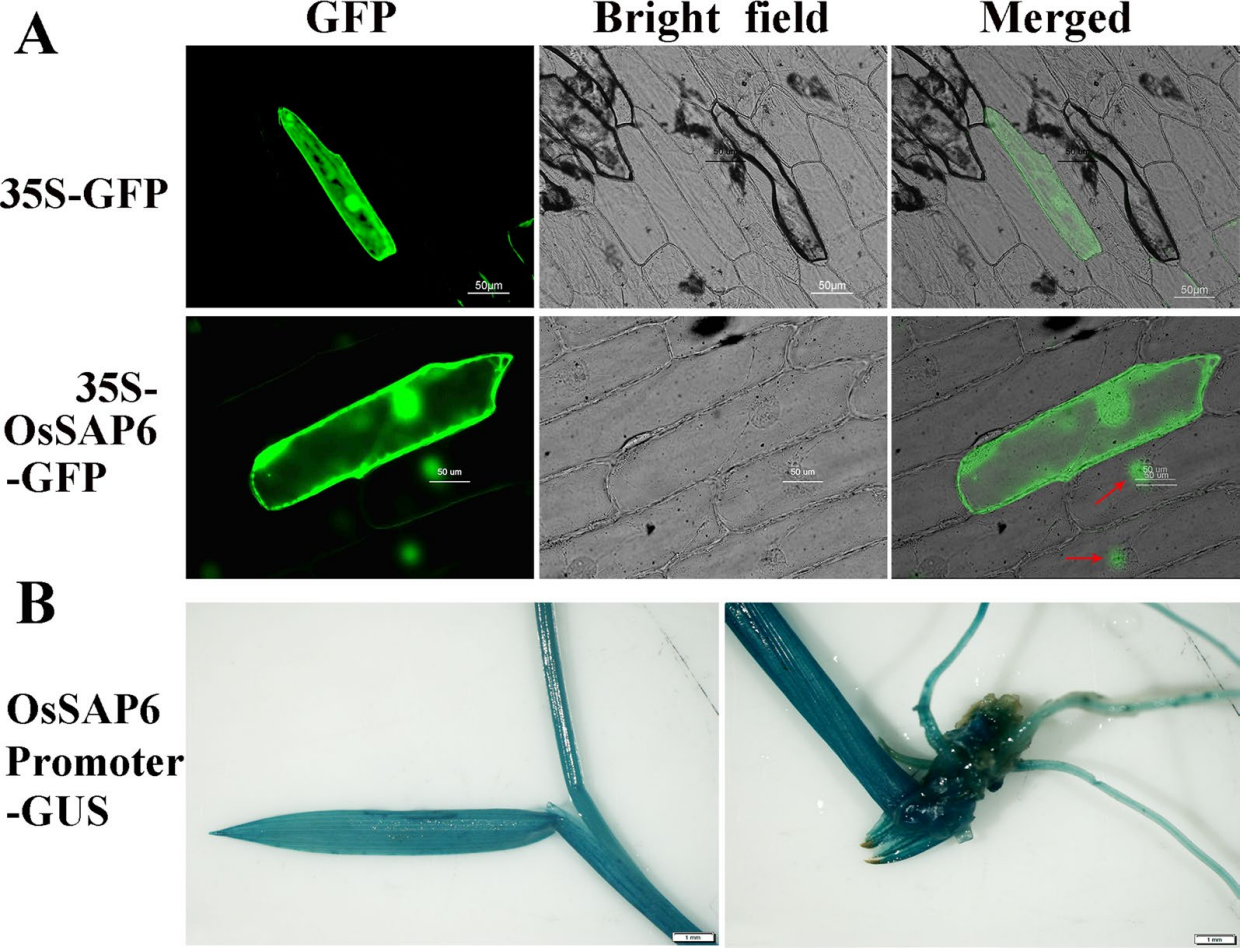
Subcellular localization of OsSAP6 in onion epidermal cells and expression of *OsSAP6* promoter-GUS. **A** GFP and OsSAP6-GFP driven by the 35S promoter under green fluorescence, bright field, and merged views. Bar, 50 μm. **B** Image of stereomicroscope after immersion in GUS stain and decolorization in 95% ethanol, Bar, 1 mm

Subcellular localization of the protein encoded by *OsSAP6* was predicted using PSORT (http://psort1.hgc.jp/form.html). The result indicated OsSAP6 may be located in the cytoplasm with a score of 0.65. The prediction scores of chloroplast stroma, chloroplast thylakoid membrane, and chloroplast thylakoid space were all 0.20. However, TargetP (https://services.healthtech.dtu.dk/service.php?TargetP-2.0) did not predict the signal peptide of the secretory pathway. The subcellular location of OsSAP6 was then experimentally verified by transient transformation of CaMV35S-*OsSAP6*-*GFP* in onion epidermis. Green fluorescent signals were detected in both cytoplasm and nucleus (Fig. 2 and Additional file 1: Fig. S3), indicating that OsSAP6-GFP was located in both cytoplasm and nucleus. In addition, a portion of the localization exhibited separate nucleus expression, speculating that intracytoplasmic localization may be caused by targeting proteins within the cytoplasm and they interacted and functioned. Although the expression differed from the software prediction, it reflected the properties of the transcription factor protein.

To understand the expression pattern of *OsSAP6* in rice, we generated transgenic lines that express a β-glucuronidase (GUS) reporter driven by the *OsSAP6* promoter. GUS staining of young seedlings of *OsSAP6* promoter-GUS transgenic lines showed blue roots, leaves, and leaf sheaths, indicating that *OsSAP6* was expressed in the whole plant.

### Overexpression of *OsSAP6* in Rice Enhances Tolerance to Soda Saline–Alkaline Stress During Germination and Vegetative Stages

*OsSAP6* was integrated into rice genome by *Agrobacterium tumefaciens* mediated genetic transformation (Additional file 1: Fig. S4A). The relative expression of *OsSAP6* in seedlings of T3 generation lines was investigated using qRT–PCR (Additional file 1: Fig. S4B). The results indicated that *OsSAP6* was overexpressed in these transgenic lines. The overexpressing lines T3-#7, 10, and 11 were selected for harvesting T3 generation seeds in preparation for subsequent experiments.

**Fig. 3:**
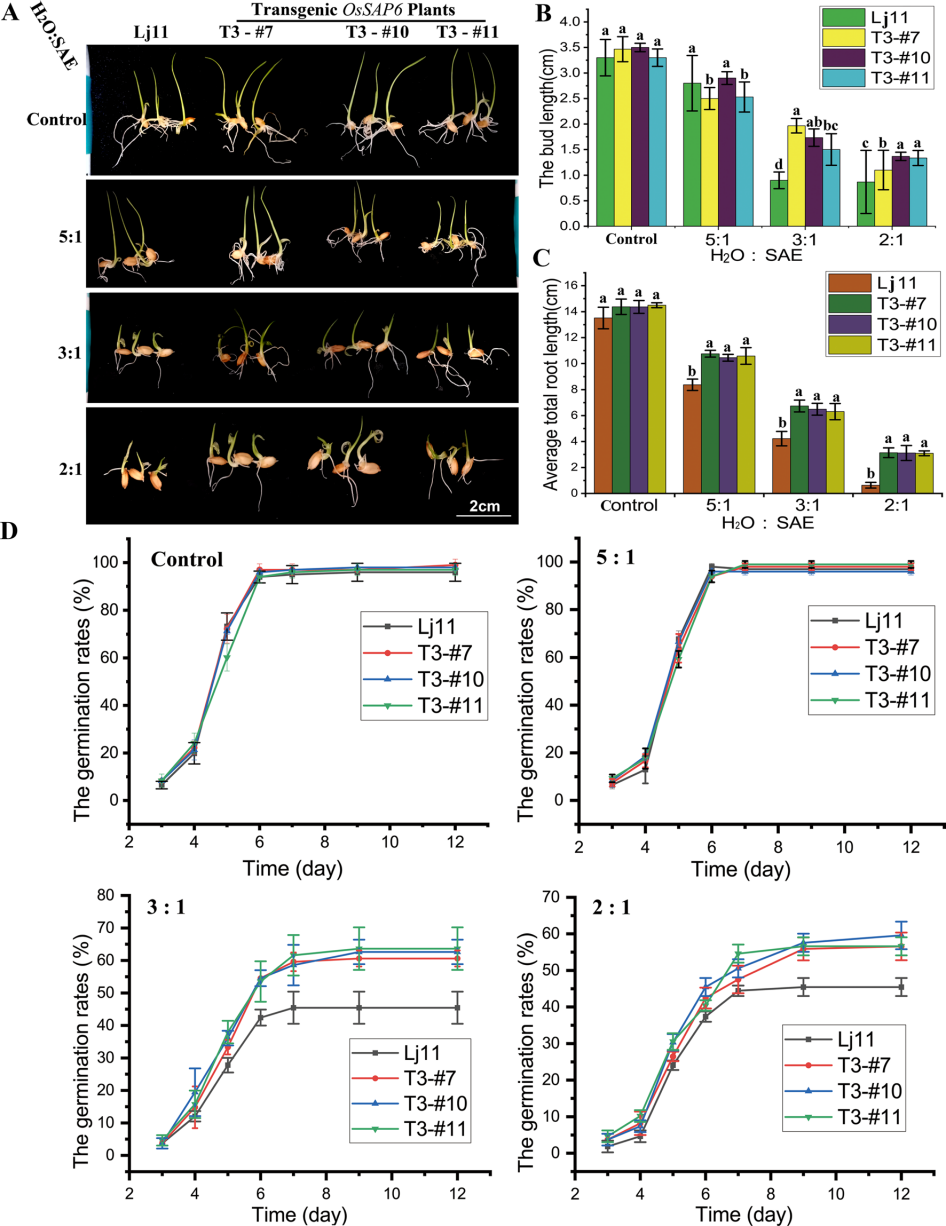
Overexpression of *OsSAP6* in rice enhanced tolerance to soda saline–alkaline stress during germination period. **A** Germination phenotypes of *OsSAP6* overexpression lines (T3-#7, 10, and 11) and Lj11 under different concentrations of SAE stress (H_2_O:SAE = 5:1, 3:1, and 2:1, water as control). **B** bud length; **C** total root length; **D** germination rate. Data show the mean ± SD of three independent replicates. At least 40 seeds per genotype were measured in each replicate. Statistical differences are labeled with different letters using LSD test (*p* < 0.05, one-way ANOVA)

We investigated germination abilities of *OsSAP6* overexpression lines and Lj11 exposed to different ratios of SAE. The results indicated that seed germination abilities of *OsSAP6* overexpression lines were better than that of the WT (Lj11) (Fig. 3A). The germination rates in water (control) and low soda saline–alkaline stress (H_2_O:SAE = 5:1) was not significant difference. However, when the stress increased to H_2_O:SAE = 3:1 and 2:1, all the germination rates decreased, but the transgenic lines were higher than that of Lj11 (Fig. 3D). The bud and total root length showed a significant difference under the high level of SAE stress. When the ratios of H_2_O:SAE were 3:1 and 2:1, the bud length of *OsSAP6* overexpression lines was approximately 1 cm longer than Lj11, and the total root length was 2–3 times longer than that of Lj11 (Fig. 3B, C). This finding suggests that the overexpression of *OsSAP6* confers tolerance to soda saline–alkaline stress during seed germination and regulates the response mechanisms to soda saline–alkaline stress.

**Fig. 4:**
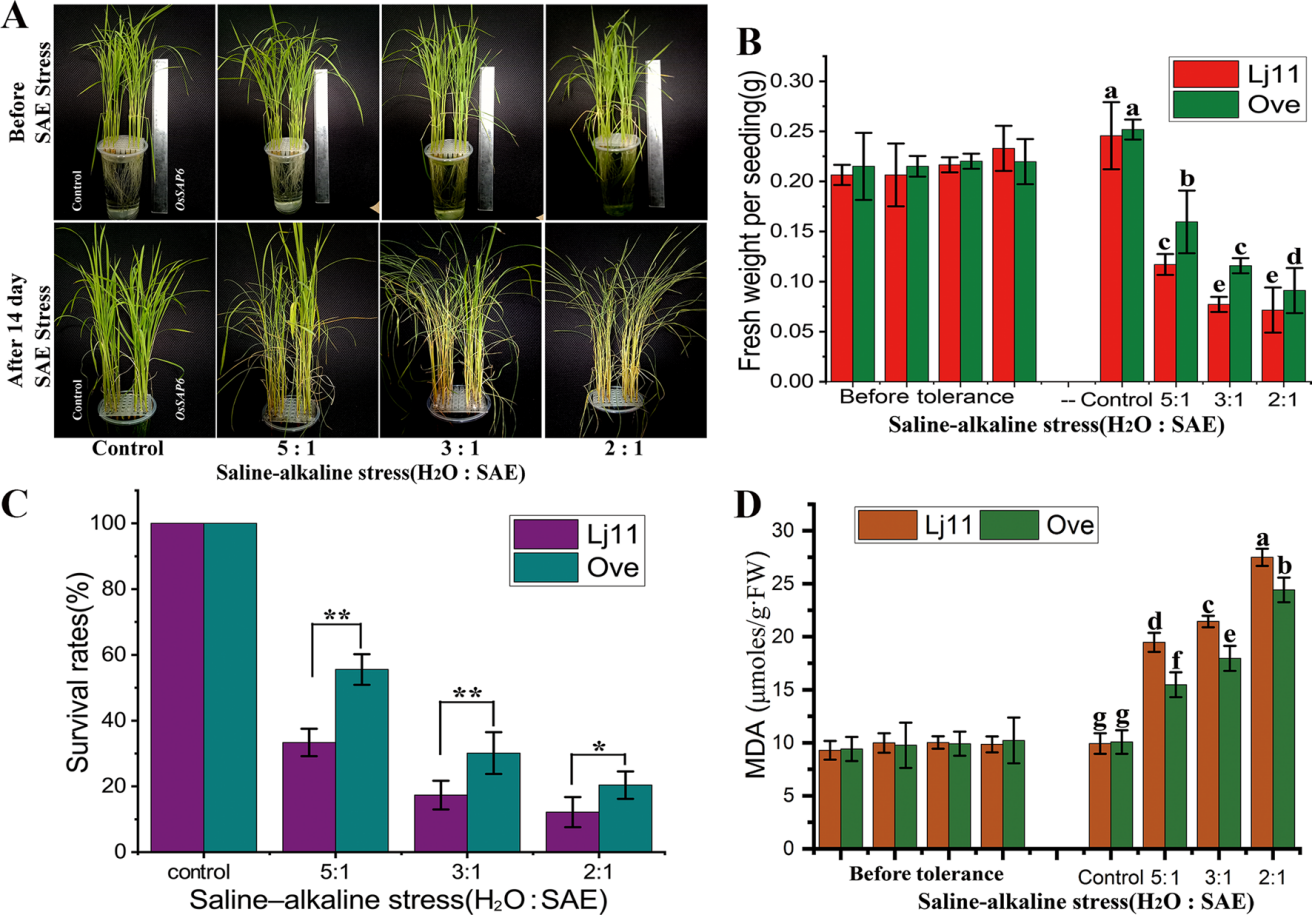
Overexpression of *OsSAP6* in rice enhances tolerance to soda saline–alkaline stress during growth period. Four-week-old Lj11 (three rows on the left) and *OsSAP6* overexpression line T3-#10 (three rows on the right) seedlings were treated with different concentrations of SAE with H_2_O:SAE = 5:1, 3:1, and 2:1, water as a control, and the growth were investigated after 14 days. **A** Phenogram of growth; **B** fresh weight of individual plants; **C** survival rate, asterisks indicate significant mean differences between *OsSAP6* overexpression lines and Lj11 (*p < 0.05 and **p < 0.01); **D** MDA contents. Data show the mean ± SD of three replicates. Statistical differences are labeled with different letters using LSD test (*p* < 0.05, one-way ANOVA)

To further investigate the molecular functions of *OsSAP6*, the overexpression lines were treated with soda saline–alkaline stress during growth period. Four-week-old *OsSAP6* overexpression line T3-#10 and Lj11 seedlings were treated with different ratios of SAE. Significant difference in growth was observed after 14 days. Lj11 had more leaves curling and wilting, and the fresh weight was significantly lower than transgenic lines (Fig. 4A, B). The survival rates of the *OsSAP6* overexpression line were higher than that of Lj11 at H_2_O:SAE = 5:1, 3:1, and 2:1 (Fig. 4C). The MDA (malondialdehyde) content were both increased under stress, but transgenic lines were significantly less than Lj11 (Fig. 4D). These results indicated that the overexpression of *OsSAP6* enhanced the tolerance to soda saline–alkaline stress in rice, and the OsSAP6 protein may be a transcription factor involved in positive regulation of alkaline salt stress.

### Relative Expression of *OsCu*/*Zn-SOD*, *OsAPX2*, and *OsCAT1* Under Soda Saline–Alkaline Stress in Transgenic Rice

**Fig. 5:**
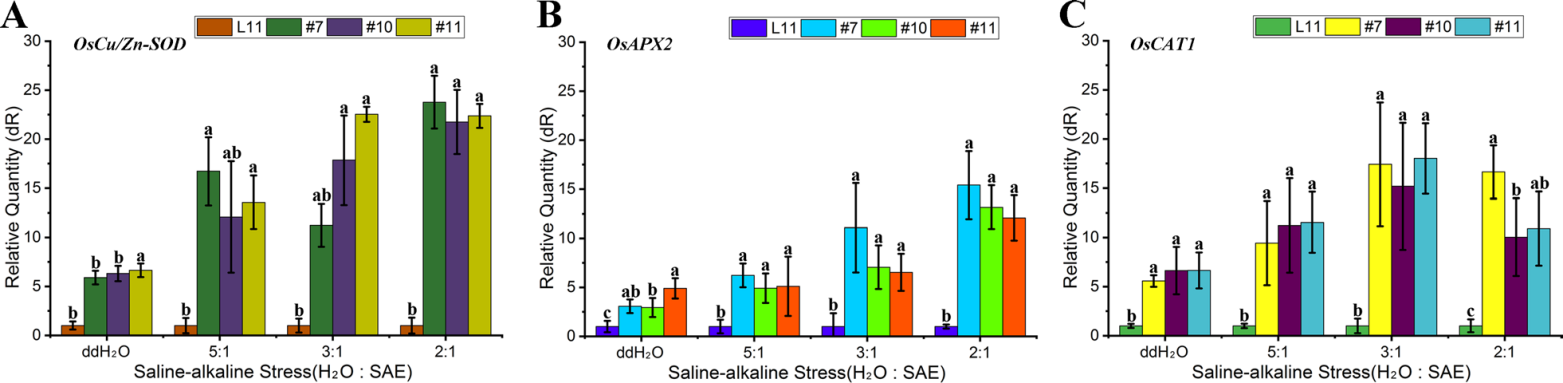
Expression of *OsCu*/*Zn-SOD*, *OsAPX2*, and *OsCAT1* in *OsSAP6* overexpression rice under soda saline–alkaline stress. 4-week-old *OsSAP6* overexpression lines and Lj11 seedlings were treated with different ratios of SAE (H_2_O:SAE = 5:1, 3:1, and 2:1, water as a control) for 7 days. The expression levels of *OsCu/Zn-SOD* (**A**), *OsAPX2* (**B**), and *OsCAT1* (**C**) were detected by qRT–PCR. The expression level of Lj11 was set to 1, and the *Os18sRNA* gene was used as an internal reference control. Values are the mean ± standard deviation of three replicates. Statistical differences are labeled with different letters using Duncan test (*p* < 0.05, one-way ANOVA)

Soda saline–alkali stress disrupts ROS balance, and the expression of genes encoding key enzymes in its scavenging pathway reflects the level of clearing excess ROS. The expression levels of *OsCu*/*Zn-SOD* in *OsSAP6* overexpression lines T3-#7, 10, and 11 were 5 times higher than that of Lj11 in the unstressed state. *OsCu/Zn-SOD* was significantly increased under SAE stress, with the transgenic lines having expression more than 20 times higher than that of Lj11 at H_2_O: SAE = 2:1. The expression of *OsAPX2* increased significantly with increasing SAE concentration and differed between lines. *OsAPX2* was increased more significantly in T3-#7 than the other lines, and increased most at H_2_O:SAE = 2:1. The expression of *OsCAT1* increased and then decreased with increasing SAE ratios, peaking at H_2_O:SAE = 3:1 and decreasing at H_2_O:SAE = 2:1, but it is still a high level relative to Lj11 (Fig. 5). Significant overexpression of the functional genes in ROS balance pathway suggests that resistance of *OsSAP6* overexpression lines under soda saline–alkaline stress may be related to the ROS balance.

### Interactional Analysis Between OsSAP6 and OsPK5

**Fig. 6:**
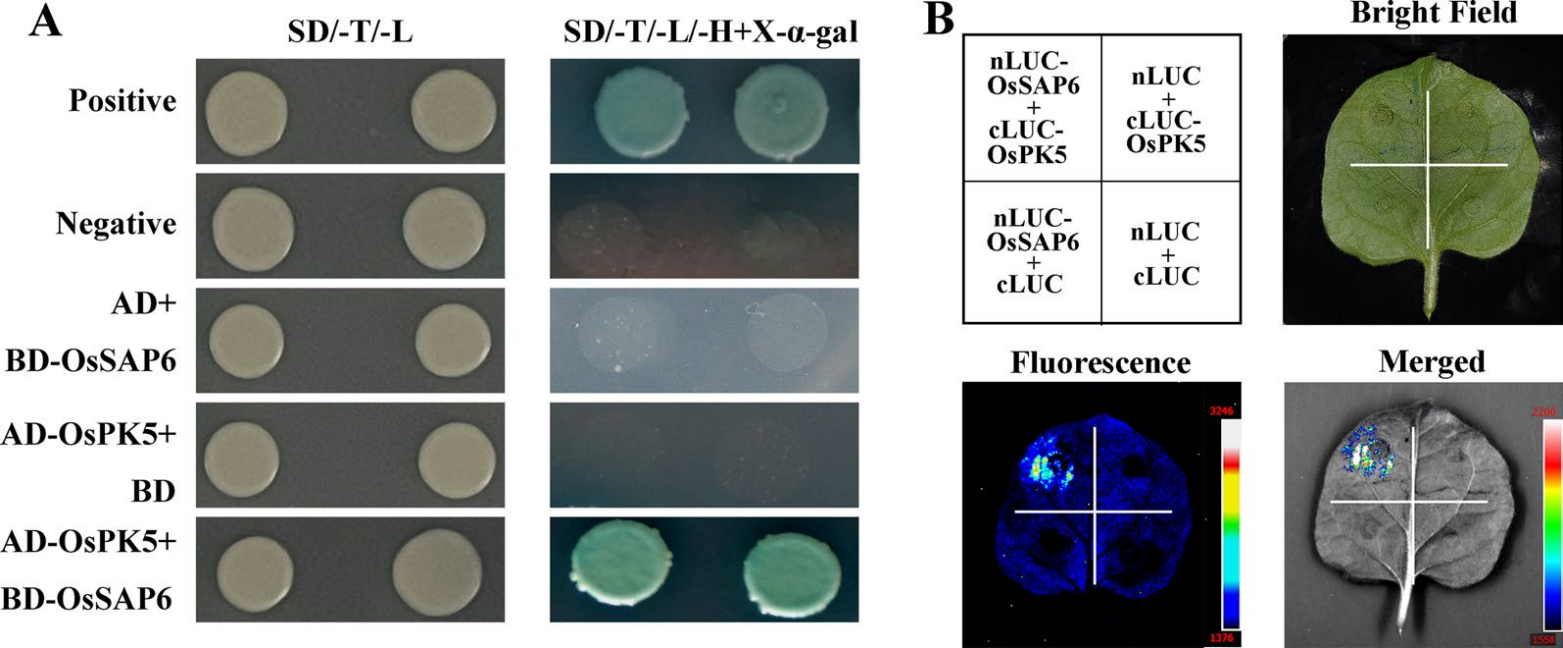
Interactional analysis between OsSAP6 and OsPK5. **A** OsSAP6 was found to interact with OsPK5 in a yeast two-hybrid assay. pGADT7-*OsPK5* was validated by cotransforming Y2HGold yeast cells with pGBKT7-*OsSAP6* plasmid, and strong interaction were found in medium SD/-Trp-Leu-His containing X‐α‐gal. **B** Interaction of OsSAP6 and OsPK5 in *N. benthamiana* leaves was analyzed by LCI assay

To identify OsSAP6 interactors we used a yeast two-hybrid system screen for a total of 16 colonies (Additional file 1: Fig. S5). According to sequencing results, *pyruvate kinase 5* (*PK5*, EU267984) was identified from the NCBI BLAST database (https://blast.ncbi.nlm.nih.gov/Blast.cgi). *OsPK5* was cloned and constructed into pGADT7 vector, then cotransformed into Y2HGold with pGBKT7-*OsSAP6* plasmids for validation. The results revealed that OsPK5 showed a strong interaction with OsSAP6 on SD/-Trp-Leu-His (synthetic drop-out medium lacking Trp, Leu, and His) containing X‐α‐gal (Fig. 6A). LCI (LUC complementation imaging) assays showed that OsSAP6 interacted with OsPK5 in *N. benthamiana* leaves (Fig. 6B), indicating that they also interacted in vivo. It is speculated that *OsSAP6* is involved in saline–alkaline stress resistance pathway in synergy with the OsPK5.

### Expression of ***OsPK5*** in Response to NaHCO_3_ and H_2_O_2_ Stress in Rice

To investigate the response of *OsPK5* to saline–alkali and oxidative stress, qRT–PCR was performed to detect the expression level of *OsPK5* in leaves and roots of Lj11 under 60 mM NaHCO_3_ and 5 mM H_2_O_2_ stress. The expression of *OsPK5* was induced upregulation by NaHCO_3_. The maximum expression of *OsPK5* in leaves and roots was 5.9 and 3.8 times higher than the control at 24 h, then slightly decreased at 48 h (Additional file 1: Fig. S6A, B). *OsPK5* responded strongly in roots under H_2_O_2_ stress, rising to 14.1 times than that of the control at 24 h. Its expression in leaves increased to 1.7 times at 6 h and then decreased (Additional file 1: Fig. S6C, D). This result indicated that *OsPK5* was responsive to NaHCO_3_ and H_2_O_2_ stress, which was speculated to be related to saline–alkaline tolerance and ROS balance.

### Overexpression of *OsPK5* in Rice Enhances Tolerance to Soda Saline–Alkaline Stress at Seedling Stage

**Fig. 7:**
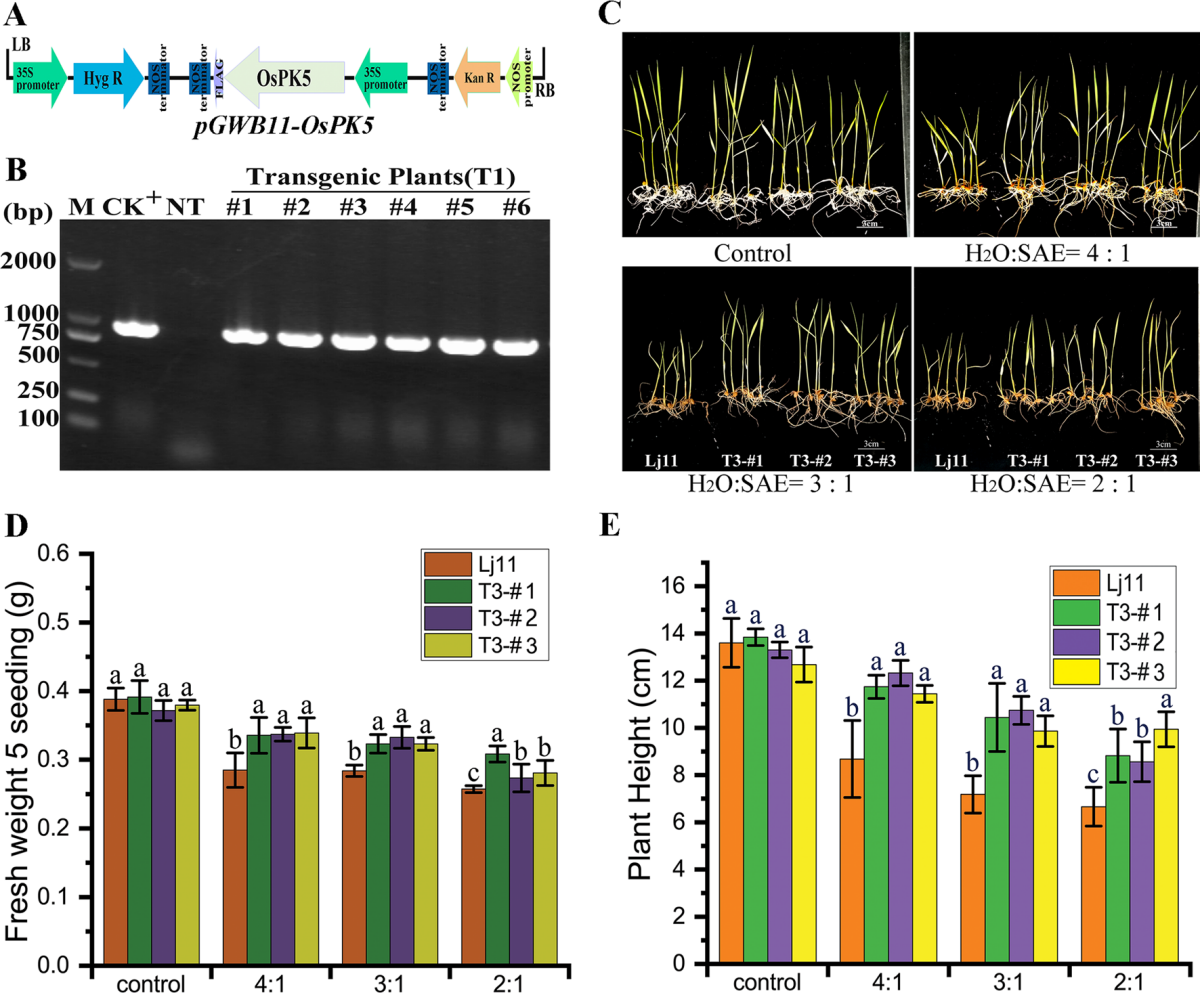
Overexpression of *OsPK5* in rice enhances tolerance to soda saline–alkaline stress at seedling stage. **A** Schematic diagram of the T-DNA insertion site in *OsPK5* overexpression lines. **B** RT–PCR detection of *OsPK5* transcripts in 6 independent transgenic lines of rice; M, Marker, DL2000; CK + , positive control, pGWB11-*OsPK5*; NT, Nontransgenic lines, Lj11. **C** Phenotypes of Lj11 and *OsPK5* overexpression lines T3-#1, 2 and 3 under SAE stress (H_2_O:SAE = 5:1, 3:1 and 2:1, water as control). **D** fresh weight of 5 seedings; **E** plant height. Data show the mean ± SD of three replicates. Statistical differences are labeled with different letters using LSD test (*p* < 0.05, one-way ANOVA)

*OsPK5* was cloned into pGWB11 vector and integrated into Lj11 genome by *Agrobacterium tumefaciens* mediated genetic transformation and detected by RT-PCR (reverse transcription PCR) (Fig. 7A, B). 7-day-old *OsPK5* overexpression lines and Lj11 seedlings were treated with SAE stress, and showed growth differences after 7 days (Fig. 7C). *OsPK5* overexpression lines T3-#1, 2, and 3 showed stronger growth and more fresh weight than Lj11 under treatments of H_2_O:SAE = 4:1 and 3:1. Although the fresh weight between lines were differences under SAE treatment of 2:1, the transgenic lines were significantly heavier than Lj11 (Fig. 7D). The plant height of the transgenic lines was significantly higher than Lj11 under stress (Fig. 7E). These results indicated that the overexpression of *OsPK5* in rice enhances the tolerance to soda saline–alkaline stress at seedling stage. The expression level of *OsCu/Zn-SOD*, *OsAPX2*, and *OsCAT1* were investigated using qRT–PCR (Additional file 1: Fig. S7). The results revealed that those ROS scavenging genes were significantly expressed in *OsPK5* overexpression lines T3-#1, 2, and 3 under different ratios of SAE treatment, indicating that *OsPK5* regulates soda saline–alkaline tolerance may be related to the ROS homeostasis.

## Discussion

Saline–alkaline stress affects plant growth and development, especially alkaline salt damage more severely. Currently, the mechanisms of plant response to biotic and abiotic stresses have become the key to the breeding of stress-resistant crop varieties (Chen et al. 2021). Studies about the resistance of *OsSAPs* have been reported in various aspects, however, there is no previous report on the resistance of *OsSAPs* under soda saline–alkaline stress. In this study, we investigated the expressional patterns of *OsSAP4*, *6*, *8*, and *9* in the same branch in response to alkaline salt NaHCO_3_ stress using qRT–PCR (Fig. 1). All these four genes were increased in both roots and leaves under NaHCO_3_ stress. The increased expression of the four genes was significantly different and *OsSAP6* was the highest one, which was increased by 8.7875 and 15.18 times in leaves and roots compared to the control at 12 h. These four genes have the same upregulated expression as most of the 18 genes of *OsSAPs* under NaCl stress. The expression of *OsSAP6* in 7-day-old rice seedlings under salt (200 mM) for 6 h was increased by about 2.7 times, and about 3.5 times of the control under dehydration for 6 h. (Vij and Tyagi 2006). The strong induction of *OsSAP6* specificity to alkaline salt in this study suggested that it might be involved in NaHCO_3_ stress response regulation. Studies have shown that the subcellular location of OsSAP1 is in nucleus. A20, AN1, and A20/AN1 of *OsSAP7* localized in nucleus of onion epidermal cells with YFP and colocalized with AtCOP1 in nucleus (Sharma et al. 2015). GFP and OsSAP8 fusion protein is localized in cytoplasm (Kanneganti and Gupta 2008), it is speculated that it may not act as a transcription factor like *OsSAP1*, because it lacks the nuclear localization sequence and DNA binding domain, and realizes its function through protein interactions (Mukhopadhyay et al. 2004). In this study, the OsSAP6-GFP fusion protein was localized in cytoplasm and nucleus (Fig. 2), speculating that it functions both as a transcription factor to bind DNA and through protein–protein interactions. OsSAP (R12H780) studied by Ubaidillah et al. (2013) is localized in mitochondria, which represents a new type of Bax suppressor-related gene and endows multiple stress tolerance in yeast.

**Fig. 8:**
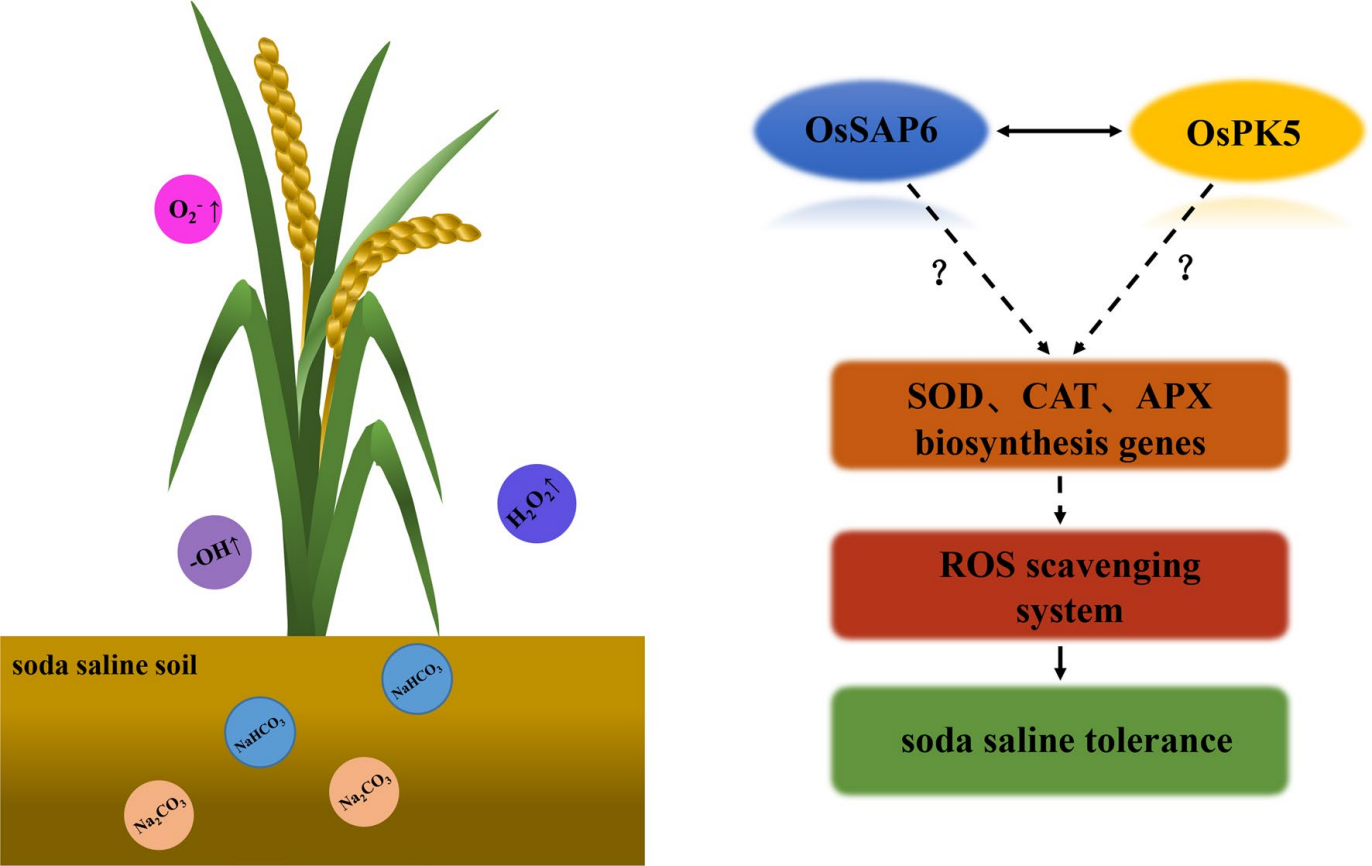
A brief pathway of OsSAP6 regulation under soda saline–alkaline stress. Overexpression of *OsSAP6* upregulates genes involved in ROS scavenging. The expression of these genes leads to a reduction in ROS accumulation, which results in improved tolerance to soda saline–alkaline stress. *OsSAP6* also interacts with *OsPK5* to positively regulate soda saline–alkaline tolerance through ROS homeostasis

The interacting protein OsPK5 was identified by Y2H assay in this study and verified by LCI analysis (Fig. 6). *OsSAP6* was hypothesized to be involved in rice physiological pathways together with *OsPK5*. Mutants with enhanced expression of *OsSAP16* have reduced stomatal conductance, which limits CO_2_ uptake and photosynthetic rate, resulting in less biomass accumulation in the mutants, and this gene is involved in modulating the response of rice to drought stress by regulating the expression of a set of stress-related genes (Wang et al. 2016). In this study, *OsSAP6* overexpression rice showed a regulatory ability to soda saline–alkaline stress tolerate during the germination period, the overexpression lines had approximately 1 cm more bud length and 2–3 times more total root length than Lj11 at H_2_O:SAE = 2:1 (Fig. 3). Seedlings of *OsSAP6* overexpression lines showed stronger tolerance than Lj11 under soda saline–alkaline stress, indicating that *OsSAP6* may positively regulate proteins or genes related to resistance to alkaline salt stress. *OsSAP6* regulates downstream genes related to ROS (Fig. 8). The reduction in MDA detected in transgenic lines also facilitates ROS homeostasis (Fig. 4). Lei et al. (2020) conducted QTL (quantitative trait locus) mapping and map-based cloning for salt tolerance at the rice bud burst stage, and the results showed that *OsSAP16* was a candidate gene of qRSL7 (quantitative trait locus related to relative shoot length on chromosome 7) for improving rice salt tolerance. This study detected an increased expression of *OsPK5* in response to soda saline–alkaline (NaHCO_3_) and H_2_O_2_ stress, echoing the effect of PKs on the metabolism of phosphoenolpyruvate and pyruvate in plastids (Weber 2004). Fourteen pyruvate kinase encoding genes have been identified in Arabidopsis with different functions (Arabidopsis Genome Initiative 2000). In this study, the soda saline–alkaline resistance analysis of *OsPK5* overexpression rice showed that *OsPK5* driven by 35S promoter improve the tolerant growth of transgenic lines, and the plant height under stress was significantly higher than Lj11 (Fig. 7), indicating that *OsPK5* is involved in the regulation of soda saline–alkaline stress resistance in rice.

## Conclusion

IN this study, we identified a *stress-associated protein 6* (*OsSAP6*) gene from rice, which was upregulated expression under NaHCO_3_ stress. Overexpression of *OsSAP6* in rice improved tolerance to soda saline–alkaline stress, and the expression of ROS-related genes was significantly upregulated in overexpression lines under SAE treatment. Moreover, we identified OsPK5 that interacts with OsSAP6, which was responsive to NaHCO_3_ and H_2_O_2_ stress, and played a positive role in soda salinity tolerance of rice. This study revealed that *OsSAP6* responds to soda saline–alkaline stress and interacts with *OsPK5* to positively regulate soda saline–alkaline tolerance through ROS homeostasis in rice.

## Materials and Methods

### Plant material and Rice Transformation

*Oryza sativa longjing11* (Lj11) seeds were donated by the research group of Qingyun Bu, Chinese Academy of Sciences. According to the rice transgenic method of Toki et al. (2006) and Upadhyaya et al. (2000), dehulled Lj11 seeds were surface-sterilized and planted on a medium supplemented with 2,4-D to induce calli. Full-length sequences of *OsSAP6* and *OsPK5* were cloned into pGWB11 vector using Gateway LR Clonase II (Invitrogen) and introduced into *Agrobacterium tumefaciens* EHA105 by electroporation to infect rice calli for transformation. The integration of the *OsSAP6* and *OsPK5* was detected by PCR with special primers (Additional file 2: Table S2). qRT–PCR was used to detect the relative expression of *OsSAP6* T3 generation transgenic rice seedlings. The seeds of the T3 generation from the overexpression lines were harvested as the material for subsequent experiments.

### Soda Saline–Alkali Soil Eluent (SAE)

This study obtained soda saline–alkaline stress using soda saline–alkali soil eluent according to the preparation method of Wang et al. (2018). At the Anda field test station, the soil of the 0–10 cm soil layer of the severe alkaline patch was dried in the shade, passed through a fine sieve of 5 mm × 5 mm, and mixed thoroughly. Then, 4 L of water was poured into 2 L of saline–alkali soil, stirred well, and left for 12 h with stirring every 4 h. Filter with filter paper to remove impurities, and the experimental SAE was obtained. The leachate’s essential characteristics used in this study are shown in Tab S3.

### Phylogenetic Analysis

The OsSAPs proteins were aligned using BioEdit software, then used for the phylogenetic analysis, and grouped by the NJ (neighbor-joining) method.

### Analysis of Gene Expression

Three-leaf stage Lj11 seedlings were treated with 60 mM NaHCO_3_ and 5 mM H_2_O_2_ (0 h treatment was the control group). Leaves and roots were taken at different periods (0 h, 6 h, 12 h, 24 h, and 48 h) to extract total RNA and reverse transcribed it into cDNA, the tenfold diluted cDNA was used as a detection template. qRT–PCR using the SYBR Green PCR master mix (TransStart) in a fluorescent quantitative PCR instrument (Agilent Mx3000p) and analyzed by the 2^−ΔΔCT^ method (Livak and Schmittgen 2001). The reaction system and procedure were described by Guan et al. (2016). All expressions were normalized against the *Os18sRNA* gene. The primers used are listed in Additional file 2: Table S2.

### Subcellular Localization

The CDS (coding sequence) of the *OsSAP6* was inserted into the pBI121-35S-*GFP* vector to construct the plant expression plasmid pBI121-35S-*OsSAP6*-*GFP*, then transformed into onion epidermal cells by a gene gun (GDS-80) and cultured for 18–24 h. The green fluorescence of OsSAP6-GFP fusion proteins was detected by scanning observation under a focusing microscope (Olympus), and the subcellular location of OsSAP6 was indicated by GFP.

### Histochemical Analysis of GUS Activity

The promoter sequence of the *OsSAP6* was identified through NCBI (LOC_Os03g57890), and primers (Additional file 2: Table S2) were designed to obtain a full length of 800 bp. The pGWB3-*OsSAP6* promoter-GUS was generated by LR reactions with the *OsSAP6* promoter driving GUS reporter gene expression. Then, transformed into Lj11 under the mediation of *Agrobacterium tumefaciens* EHA105, and the GUS activity was detected by staining with X-Gluc. The samples were destained with 95% ethanol and then examined.

### Soda Saline–Alkaline Stress Tolerance Assay

Full-grown seeds were surface-sterilized and treated with different ratios of SAE including H_2_O:SAE = 5:1 (5 ml H_2_O + 1 ml SAE), 3:1 (4.5 ml H_2_O + 1.5 ml SAE), 2:1 (4 ml H_2_O + 2 ml SAE), and control (6 ml H_2_O). Seed germination was defined as the germ reaching half the length of the seed. The germination rate was calculated from the 3rd day, recorded every 24 h for 12 d, and detected the bud length and the total root length. To test the tolerance of transgenic lines to soda saline–alkaline stress, T3 generation transgenic lines T3-#10 and Lj11 were grown in 1/8 MS nutrient solution for 4 weeks, then stressed in different ratios of SAE with a total volume of 600 ml included 5:1, 3:1, 2:1 and the control. The phenotype, survival rates, and fresh weight were investigated after 14 days.

Seeds of overexpressing *OsPK5* lines and Lj11 were germinated for 7 days, soaked in different ratios of H_2_O to SAE (4:1, 3:1, 2:1, water as control) for 7 days, and investigated for phenotype, fresh weight per plant and plant height.

To detect the expression of salinity-related genes, *OsCu*/*Zn-SOD*, *OsAPX2*, and *OsCAT1* gene expression in transgenic lines under SAE stress were detected by qRT–PCR. The primers are shown in Additional file 2: Table S2.

### Yeast Two-Hybrid Assay

The full-length ORF (open reading frame) sequence of *OsSAP6* was fused into pGBKT7 vector as the bait and transformed into the yeast strain Y2HGold. Then cocultured with yeast containing cDNA from the japonica cultivar *Nipponbare* library (pGADT7-*Rice*) on SD/-Trp-Leu-His, SD/‐Ade-His-Leu-Trp, and SD/‐Trp‐Leu containing X‐α‐gal, and the blue strains were preserved (the experiment was repeated n = 3). The gene of the interacting protein was cloned with the primer T7/3-AD (Additional file 2: Table S2), and the interaction factor was obtained by sequencing. To validate the interaction, we searched for interacting factors in the NCBI Gene Bank according to the sequencing results, designed primers (Additional file 2: Table S2) based on the mRNA sequence and cloned them into the pGADT7 vector. Then cotransformed into Y2HGold with pGBKT7-*OsSAP6* and cultured on synthetic drop-out medium SD/-His-Leu-Trp containing X‐α‐gal. The colonies with interactions were blue; otherwise, they were false-positive clones.

### LCI Assays

The OsSAP6 coding sequence was inserted into pCAMBIA1300-nLUC vector, and the full-length OsPK5 sequence was inserted into pCAMBIA1300-cLUC vector using a seamless cloning and assembly kit, then electroporated into *Agrobacterium tumefaciens* strain EHA105. Agrobacterium containing pCAMBIA1300-nLUC-*OsSAP6* was coinjected with Agrobacterium containing pCAMBIA1300-cLUC-*OsPK5* into the same part of the *N. benthamiana* leaf and incubated for 48 h. LUC activity after luciferin injection was analyzed using chemiluminescence imaging (Tanon 5200).

### Data Statistical Analysis Methods

Statistical analysis was carried out using Microsoft Excel 2013, SPSS 12.5, and DPS system for Windows, and one-way analysis of variance (ANOVA) was used for analysis. Statistical significance was defined as *p* < 0.05.

## Supplementary Information

**Additional file 1: Figure S1.** Homology analysis of OsSAP proteins. The amino acid sequence homology of the 18 OsSAP proteins was aligned by BioEdit software. **Figure S2.** Phylogenetic analysis of OsSAP proteins. The amino acid sequence homology of 18 OsSAP proteins was grouped by the neighbor-joining method phylogenetic analysis results, a bootstrap method with 1,000 replications was used for test of phylogeny. Scale bar indicates 0.2 amino acid substitution per site. **Figure S3.** Subcellular localization of OsSAP6-GFP at other excitation wavelengths. OsSAP6-GFP were observed at blue excitation group (383nm), red excitation group (587nm) and merge of green and red excitation group. Bar, 50 μm. **Figure S4.** Identification of *OsSAP6* overexpression lines. **A** The integration of *OsSAP6* into the genome of Lj11 in *OsSAP6*-overexpressing lines T0 was detected by PCR with specific primers. **B** The relative expression of *OsSAP6* in seedlings of T3 generation transformed lines was measured using qRT–PCR. The values of Lj11 are set as 1. Values are mean ± SD; n = 3. Statistical analyses were performed using Student’s t test: *p < 0.05, **p < 0.01. **Figure S5.** Colonies interacting with OsSAP6 protein in the rice cDNA library. To identify the interactors of the OsSAP6 protein, a yeast two-hybrid system was used to preliminarily screen out a total of 16 colonies that turned blue on SD/‐Trp‐Leu-His containing X‐α‐gal. Four genes were identified according to Y2H assay, namely *2-phospho-D-glycerate hydrolase* (*PGH2*, AK099342), *voltage-dependent anion channel* (*VDAC1*, AK071833), *pyruvate kinase 5 *(*PK5*, EU267984) and *probable plastid-lipid-associated protein 2* (*PAP2*, AK104742). These proteins include proteins responsible for regulating ion channels and enzymes, and may cooperate with OsSAP6 proteins to participate in abiotic stress response mechanisms. **Figure S6.** Expression of *OsPK5* in response to NaHCO_3_ and H_2_O_2_ stress in rice. qRT–PCR detected *OsPK5* expression in leaves and roots of Lj11 treated with 60 mM NaHCO_3_ and 5 mM H_2_O_2_ stress. The values of 0 h (control) are set as 1. Values are mean ± SD; n = 3. Statistical analyses were performed using Student’s t test: *p < 0.05, **p < 0.01. **Figure S7.** Expression of *OsCu*/*Zn-SOD*, *OsAPX2* and *OsCAT1* in *OsPK5* overexpression rice under soda saline-alkaline stress. 7-day-old *OsPK5* overexpression lines and Lj11 seedlings were treated with different ratios of SAE (H_2_O:SAE = 4:1, 3:1, and 2:1, water as a control) for 7 days. The expression levels of *OsCu/Zn-SOD*, *OsAPX2* and *OsCAT1* were detected by qRT–PCR. The expression level of Lj11 was set to 1, and the *Os18sRNA* gene was used as an internal reference control. Values are the mean ± standard deviation of three replicates. Statistical differences are labeled with different letters using Duncan test (*p* < 0.05, one-way ANOVA)

**Additional file 2: Table S1.** Distribution of OsSAP gene family encoding A20/AN1 zinc-Finger proteins in the rice Genes. **Table S2.** Primers used in this study. **Table S3.** The leachate’s essential characteristics.

## Data Availability

All data supporting the findings of this study are available within the paper and within its supplementary materials published online.

## Abbreviations

SAP: Stress-associated protein
Lj11: Oryza sativa longjing11
SAE: Soda saline–alkali soil eluent
ABA: Abscisic acid
ROS: Reactive oxygen species
HSP90C: Chloroplast heat shock protein 90
DRIP: Dehydration-responsive element binding protein2a interacting protein
MBP-1: C-myc binding protein
qRT–PCR: Quantitative real time PCR
Y2H: Yeast two hybrid
GUS: β-Glucuronidase
MDA: Malondialdehyde
SD: Synthetic drop-out medium
LCI: LUC complementation imaging
RT-PCR: Reverse transcription PCR
QTL: Quantitative trait locus
qRSL7: Quantitative trait locus related to relative shoot length on chromosome 7
NJ: Neighbor-joining
CDS: Coding sequence
ORF: Open reading frame

## References

[CR1] Arabidopsis Genome Initiative (2000). Analysis of the genome sequence of the flowering plant Arabidopsis thaliana. Nature.

[CR2] Ben Saad R, Ben Ramdhan W, Zouari N, Azaza J, Mieulet D, Guiderdoni E, Ellouz R, Hassairi A (2012). Marker-free transgenic durum wheat cv. Karim expressing the AlSAP gene exhibits a high level of tolerance to salinity and dehydration stresses. Mol Breed.

[CR3] Ben Saad R, Fabre D, Mieulet D, Meynard D, Dingkuhn M, Al-Doss A, Guiderdoni E, Hassairi A (2012). Expression of the Aeluropus littoralis AlSAP gene in rice confers broad tolerance to abiotic stresses through maintenance of photosynthesis. Plant Cell Environ.

[CR4] Chen X, Ding Y, Yang Y, Song C, Wang B, Yang S, Guo Y, Gong Z (2021). Protein kinases in plant responses to drought, salt, and cold stress. J Integr Plant Biol.

[CR5] Dansana PK, Kothari KS, Vij S, Tyagi AK (2014). OsiSAP1 overexpression improves water-deficit stress tolerance in transgenic rice by affecting expression of endogenous stress-related genes. Plant Cell Rep.

[CR6] Dixit AR, Dhankher OP (2011). A novel stress-associated protein 'AtSAP10' from Arabidopsis thaliana confers tolerance to nickel, manganese, zinc, and high temperature stress. PLoS ONE.

[CR7] Dreher K, Callis J (2007). Ubiquitin, hormones and biotic stress in plants. Ann Bot.

[CR8] Evans PC, Ovaa H, Hamon M, Kilshaw PE, Hamm S, Bauer S, Ploegh HL, Smith TS (2004). Zinc-finger protein A20, a regulator of inflammation and cell survival, has de-ubiquitinating activity. Biochem J.

[CR9] Gimeno-Gilles C, Gervais ML, Planchet E, Satour P, Limami AM, Lelievre E (2011). A stress-associated protein containing A20/AN1 zinc-finger domains expressed in *Medicago truncatula* seeds. Plant Physiol Biochem.

[CR10] Giri J, Vij S, Dansana PK, Tyagi AK (2011). Rice A20/AN1 zinc-finger containing stress-associated proteins (SAP1/11) and a receptor-like cytoplasmic kinase (OsRLCK253) interact via A20 zinc-finger and confer abiotic stress tolerance in transgenic Arabidopsis plants. New Phytol.

[CR11] Guan QJ, Ma HY, Wang ZJ, Wang ZY, Bu QY, Liu SK (2016). A rice LSD1-like-type ZFP gene OsLOL5 enhances saline–alkaline tolerance in transgenic Arabidopsis thaliana, yeast and rice. BMC Genomics.

[CR12] Hellmann H, Hobbie L, Chapman A, Dharmasiri S, Dharmasiri N, del Pozo C, Reinhardt D, Estelle M (2003). Arabidopsis AXR6 encodes CUL1 implicating SCF E3 ligases in auxin regulation of embryogenesis. EMBO J.

[CR13] Huang J, Teng L, Li L, Liu T, Li L, Chen D, Xu LG, Zhai Z, Shu HB (2004). ZNF216 is an A20-like and IkappaB kinase gamma-interacting inhibitor of NFkappaB activation. J Biol Chem.

[CR14] Huang J, Wang MM, Jiang Y, Bao YM, Huang X, Sun H, Xu DQ, Lan HX, Zhang HS (2008). Expression analysis of rice A20/ AN1-type zinc finger genes and characterization of ZFP177 that contributes to temperature stress tolerance. Gene.

[CR15] Kang M, Fokar M, Abdelmageed H, Allen RD (2011). Arabidopsis SAP5 functions as a positive regulator of stress responses and exhibits E3 ubiquitin ligase activity. Plant Mol Biol.

[CR16] Kanneganti V, Gupta AK (2008). Overexpression of OsiSAP8, a member of stress associated protein (SAP) gene family of rice confers tolerance to salt, drought and cold stress in transgenic tobacco and rice. Plant Mol Biol.

[CR17] Kelley DR, Estelle M (2012). Ubiquitin-mediated control of plant hormone signaling. Plant Physiol.

[CR18] Lee HK, Cho SK, Son O, Xu Z, Hwang I, Kim WT (2009). Drought stress-induced Rma1H1, a RING membrane-anchor E3 ubiquitin ligase homolog, regulates aquaporin levels via ubiquitination in transgenic Arabidopsis plants. Plant Cell.

[CR19] Lei L, Zheng H, Bi Y, Yang L, Liu H, Wang J, Sun J, Zhao H, Li X, Li J, Lai Y, Zou D (2020). Identification of a major QTL and candidate gene analysis of salt tolerance at the bud burst stage in rice (*Oryza sativa* L.) using QTL-Seq and RNA-Seq. Rice (n Y).

[CR20] Li X, Cai W, Zhang S, Xu L, Chen P, Wang J (2011). Cloning and expression pattern of a zinc finger protein gene ShSAP1 in *Saccharum officinarum*. Sheng Wu Gong Cheng Xue Bao.

[CR21] Liu L, Wang B (2021). Protection of halophytes and their uses for cultivation of saline–alkali Soil in China. Biology (basel).

[CR22] Livak KJ, Schmittgen TD (2001). Analysis of relative gene expression data using real-time quantitative PCR and the 2(-Delta Delta C(T)) method. Methods.

[CR23] Lyzenga WJ, Stone SL (2012). Abiotic stress tolerance mediated by protein ubiquitination. J Exp Bot.

[CR24] Mukhopadhyay A, Vij S, Tyagi AK (2004). Overexpression of a zinc-finger protein gene from rice confers tolerance to cold, dehydration, and salt stress in transgenic tobacco. Proc Natl Acad Sci USA.

[CR25] Sharma G, Giri J, Tyagi AK (2015). Rice OsiSAP7 negatively regulates ABA stress signalling and imparts sensitivity to water-deficit stress in Arabidopsis. Plant Sci.

[CR26] Singh K, Foley RC, Oñate-Sánchez L (2002). Transcription factors in plant defense and stress responses. Curr Opin Plant Biol.

[CR27] Sreedharan S, Shekhawat UK, Ganapathi TR (2012). MusaSAP1, a A20/AN1 zinc finger gene from banana functions as a positive regulator in different stress responses. Plant Mol Biol.

[CR28] Ströher E, Wang XJ, Roloff N, Klein P, Husemann A, Dietz KJ (2009). Redox-dependent regulation of the stress-induced zinc-finger protein SAP12 in *Arabidopsis thaliana*. Mol Plant.

[CR29] Toki S, Hara N, Ono K, Onodera H, Tagiri A, Oka S, Tanaka H (2006). Early infection of scutellum tissue with Agrobacterium allows high-speed transformation of rice. Plant J.

[CR30] Ubaidillah M, Kim KA, Kim YH, Lee IJ, Yun BW, Kim DH, Loake GJ, Kim KM (2013). Identification of a drought-induced rice gene, OsSAP, that suppresses Bax-induced cell death in yeast. Mol Biol Rep.

[CR31] Upadhyaya NM, Surin R, Ramm K, Gaudron J, Schünmann PHD, Taylor W (2000). Agrobacterium mediated transformation of Australian rice cultivars Jarrah and Amaroo using modified promoters and selectable markers. Plant Physiol.

[CR32] Vij S, Tyagi AK (2006). Genome-wide analysis of the stress associated protein (SAP) gene family containing A20/AN1 zinc-finger(s) in rice and their phylogenetic relationship with Arabidopsis. Mol Genet Genomics.

[CR33] Wang F, Coe RA, Karki S, Wanchana S, Thakur V, Henry A, Lin HC, Huang J, Peng S, Quick WP (2016). Overexpression of OsSAP16 regulates photosynthesis and the expression of a broad range of stress response genes in rice (*Oryza sativa* L.). PLoS ONE.

[CR34] Wang H, Takano T, Liu S (2018). Screening and evaluation of saline–alkaline tolerant germplasm of rice (*Oryza sativa* L.) in soda saline–alkali soil. Agronomy.

[CR35] Weber AP (2004). Solute transporters as connecting elements between cytosol and plastid stroma. Curr Opin Plant Biol.

[CR36] Xuan N, Liu X, Zhang H, Yang Y, Yao F (2015). Sequence analysis of maize zinc- finger protein gene ZmAN11 and its expression status under abiotic stress. Shandong Agric Sci.

[CR37] Xuan N, Jin Y, Zhang H, Xie Y, Liu Y, Wang G (2011). A putative maize zinc-finger protein gene, ZmAN13, participates in abiotic stress response. Plant Cell Tissue Organ Cult.

[CR38] Yun X, Chen Y (2020). International development of saline–alkali land and its enlightenment to China. Territ Nat Resour Study.

[CR39] Zhang N, Xu J, Liu X, Liang W, Xin M, Du J, Hu Z, Peng H, Guo W, Ni Z, Sun Q, Yao Y (2019). Identification of HSP90C as a substrate of E3 ligase TaSAP5 through ubiquitylome profiling. Plant Sci.

[CR40] Zhang N, Yin Y, Liu X, Tong S, Xing J, Zhang Y, Pudake RN, Izquierdo EM, Peng H, Xin M, Hu Z, Ni Z, Sun Q, Yao Y (2017). The E3 Ligase TaSAP5 Alters Drought Stress Responses by Promoting the Degradation of DRIP Proteins. Plant Physiol.

[CR41] Zhang X, Wang N (2016). The development and management of saline–alkali land in Heilongjiang Province. Cognit Pract.

[CR42] Zhu JF, Cui ZR, Wu CH, Deng C, Chen JH, Zhang HX (2018). Research advances and prospect of saline and alkali land greening in China. World Forestry Research.

