# Supplementary material for: OsSAP6 Positively Regulates Soda Saline–Alkaline Stress Tolerance in Rice
Source: Rice (N Y). 2022 Dec 27;15:69. doi: 10.1186/s12284-022-00616-x (PMC9794665; doi:10.1186/s12284-022-00616-x)
Supplement: Supplementary file 1 — Additional file 1: Figure S1. Homology analysis of OsSAP proteins. The amino acid sequence homology of the 18 OsSAP proteins was aligned by BioEdit software. Figure S2. Phylogenetic analysis of OsSAP proteins. The amino acid sequence homology of 18 OsSAP proteins was grouped by the neighbor-joining method phylogenetic analysis results, a bootstrap method with 1,000 replications was used for test of phylogeny. Scale bar indicates 0.2 amino acid substitution per site. Figure S3. Subcellular localization of OsSAP6-GFP at other excitation wavelengths. OsSAP6-GFP were observed at blue excitation group (383nm), red excitation group (587nm) and merge of green and red excitation group. Bar, 50 μm. Figure S4. Identification of OsSAP6 overexpression lines. A The integration of OsSAP6 into the genome of Lj11 in OsSAP6-overexpressing lines T0 was detected by PCR with specific primers. B The relative expression of OsSAP6 in seedlings of T3 generation transformed lines was measured using qRT–PCR. The values of Lj11 are set as 1. Values are mean ± SD; n = 3. Statistical analyses were performed using Student’s t test: *p < 0.05, **p < 0.01. Figure S5. Colonies interacting with OsSAP6 protein in the rice cDNA library. To identify the interactors of the OsSAP6 protein, a yeast two-hybrid system was used to preliminarily screen out a total of 16 colonies that turned blue on SD/‐Trp‐Leu-His containing X‐α‐gal. Four genes were identified according to Y2H assay, namely 2-phospho-D-glycerate hydrolase (PGH2, AK099342), voltage-dependent anion channel (VDAC1, AK071833), pyruvate kinase 5 (PK5, EU267984) and probable plastid-lipid-associated protein 2 (PAP2, AK104742). These proteins include proteins responsible for regulating ion channels and enzymes, and may cooperate with OsSAP6 proteins to participate in abiotic stress response mechanisms. Figure S6. Expression of OsPK5 in response to NaHCO3 and H2O2 stress in rice. qRT–PCR detected OsPK5 expression in leaves and roots of Lj11 treated w [file 12284_2022_616_MOESM1_ESM.docx]

**Additional file 1: Supplemental Figures**


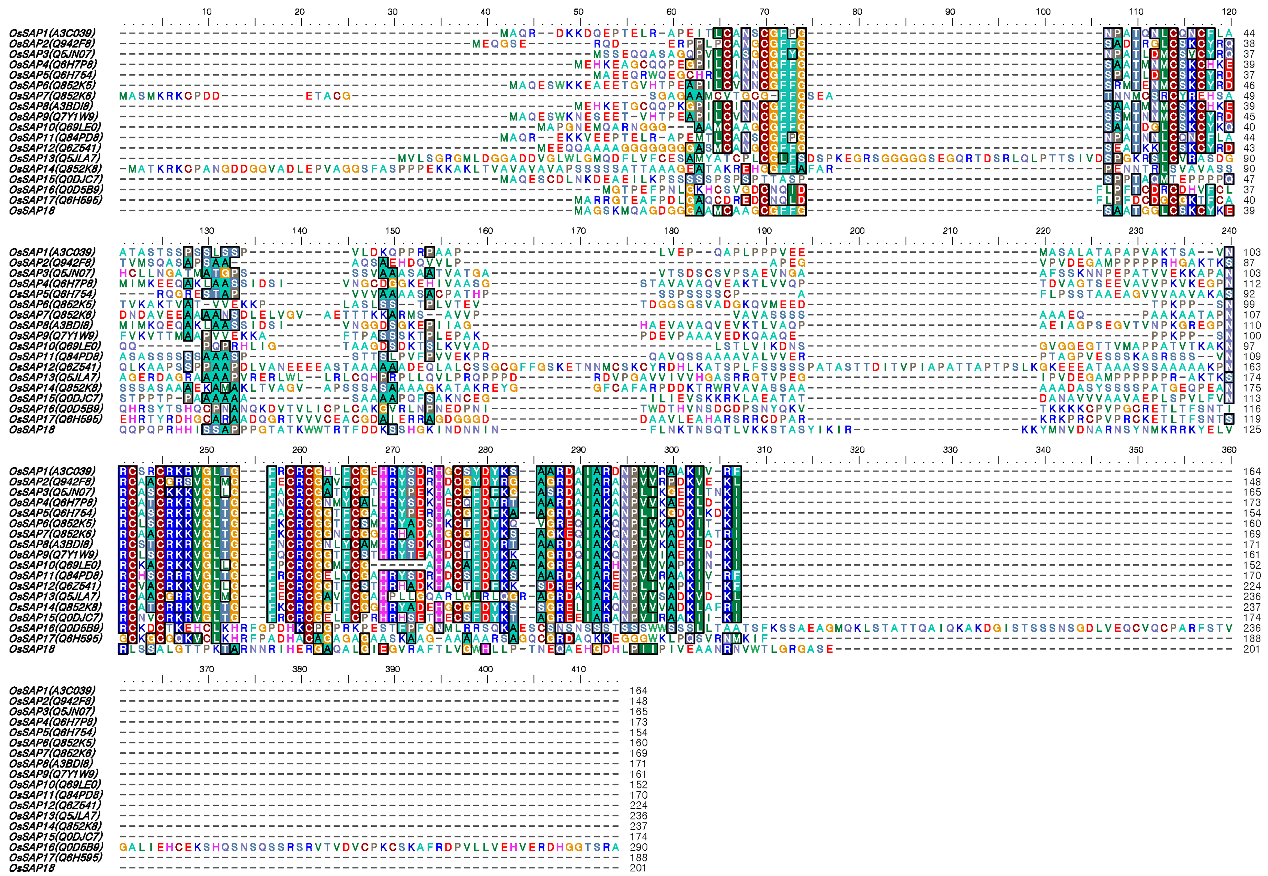


**Figure S1. Homology analysis of OsSAP proteins**

The amino acid sequence homology of the 18 OsSAP proteins was aligned by BioEdit software.





**Figure S2. Phylogenetic analysis of OsSAP proteins**

The amino acid sequence homology of 18 OsSAP proteins was grouped by the neighbor-joining method phylogenetic analysis results, a bootstrap method with 1,000 replications was used for test of phylogeny. Scale bar indicates 0.2 amino acid substitution per site.


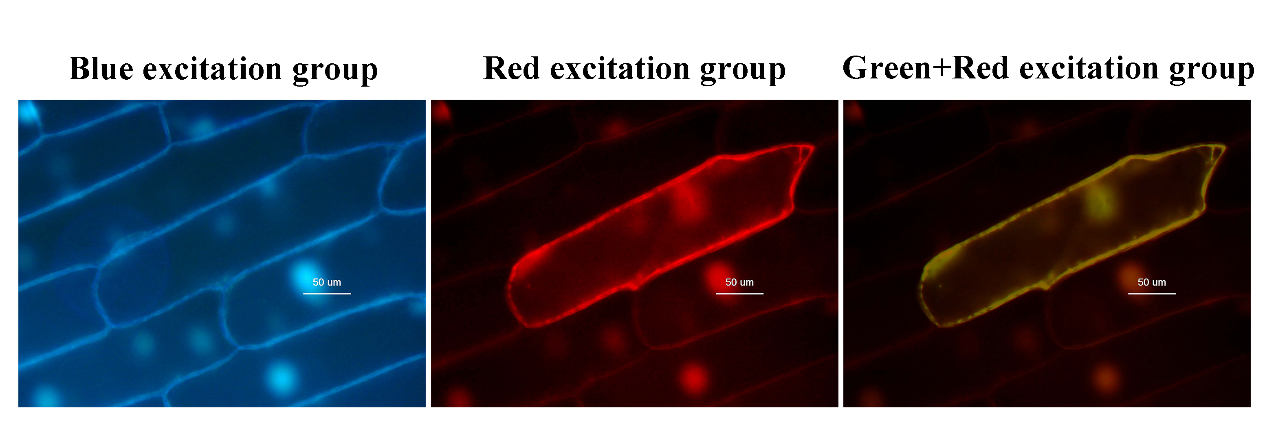


**Figure S3.** **Subcellular localization of OsSAP6-GFP at other** **excitation wavelengths**

OsSAP6-GFP were observed at blue excitation group (383nm), red excitation group (587nm) and merge of green and red excitation group. Bar, 50 μm.

**
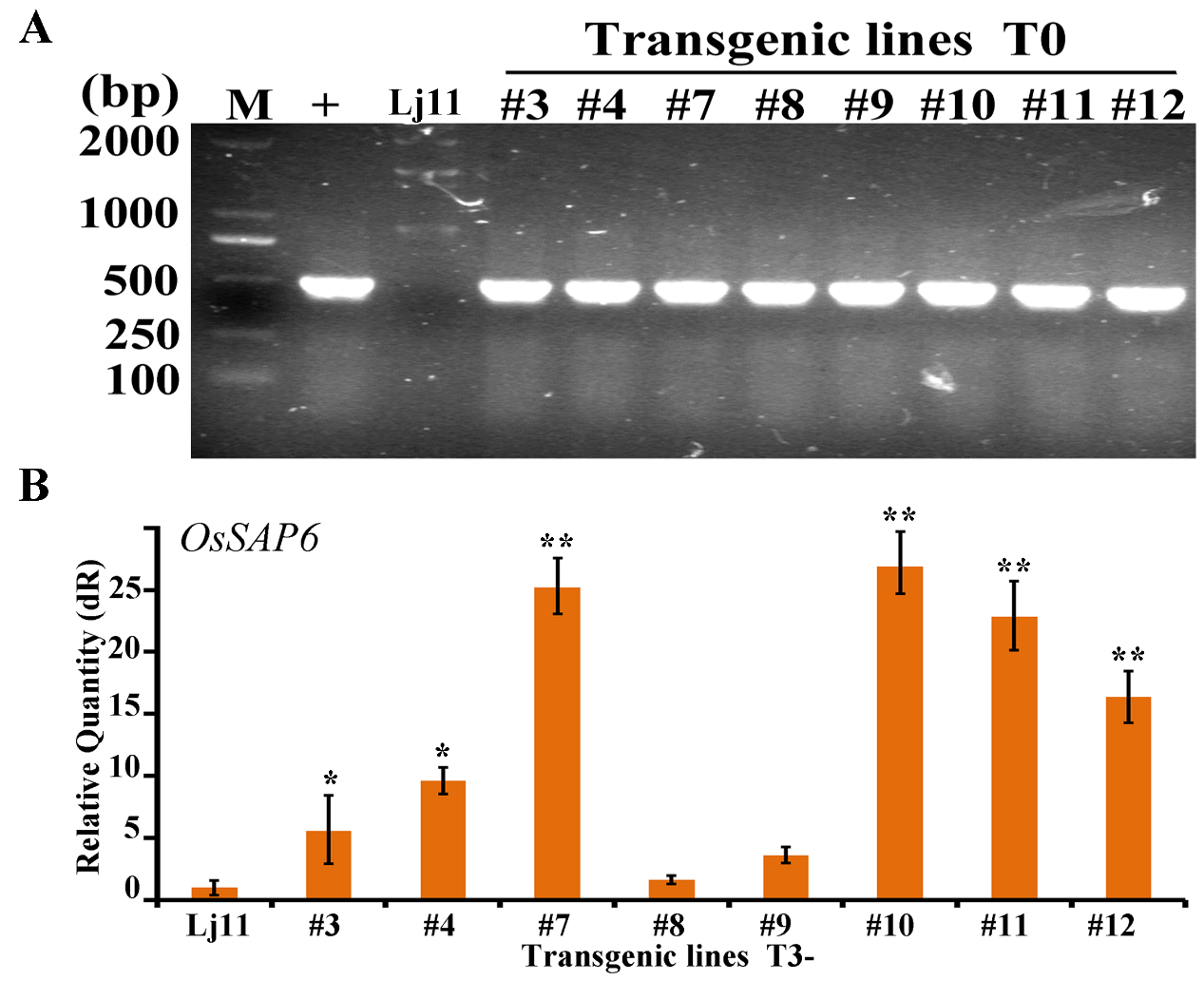
Figure S4. Identification of *OsSAP6* overexpression lines.**

A. The integration of *OsSAP6* into the genome of Lj11 in *OsSAP6*-overexpressing lines T0 was detected by PCR with specific primers. B. The relative expression of *OsSAP6* in seedlings of T3 generation transformed lines was measured using qRT–PCR. The values of Lj11 are set as 1. Values are mean ± SD; n = 3. Statistical analyses were performed using Student’s t test: **p* < 0.05, ***p* < 0.01.


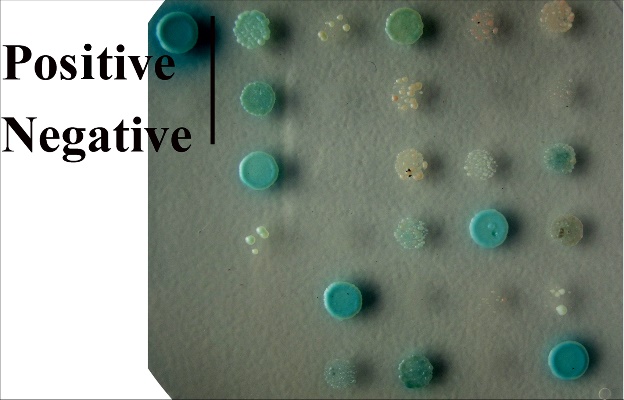


**Figure S5.** **Colonies interacting with OsSAP6 protein in the rice cDNA library**

To identify the interactors of the OsSAP6 protein, a yeast two-hybrid system was used to preliminarily screen out a total of 16 colonies that turned blue on SD/‐Trp‐Leu-His containing X‐α‐gal. Four genes were identified according to Y2H assay, namely *2-phospho-D-glycerate hydrolase* (*PGH2*, AK099342), *voltage-dependent anion channel* (*VDAC1*, AK071833), *pyruvate kinase 5* (*PK5*, EU267984) and *probable plastid-lipid-associated protein 2* (*PAP2*, AK104742). These proteins include proteins responsible for regulating ion channels and enzymes, and may cooperate with OsSAP6 proteins to participate in abiotic stress response mechanisms.


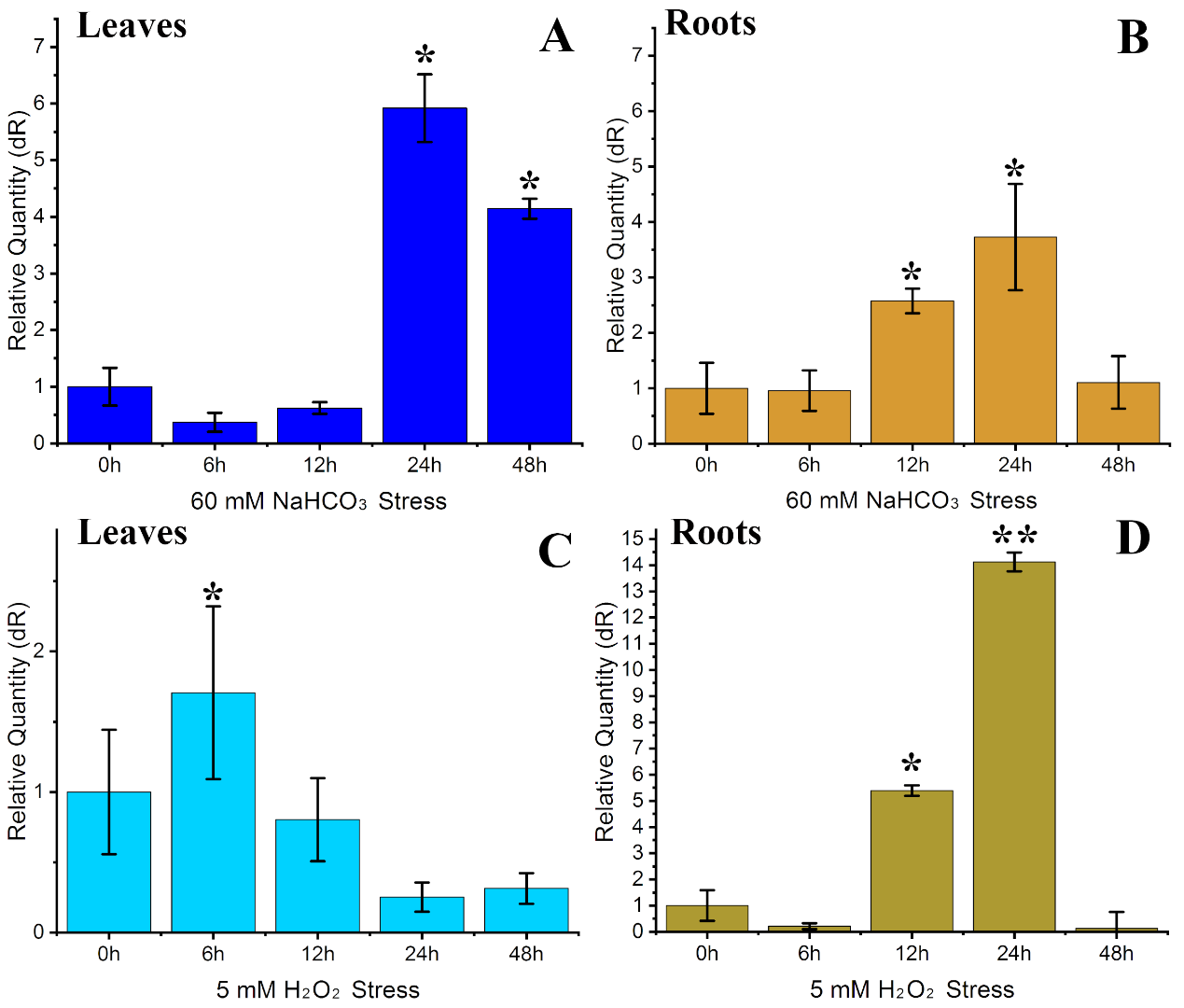


**Figure S6.** **Expression of *OsPK5* in response to NaHCO_3_ and H_2_O_2_ stress in rice**

qRT–PCR detected *OsPK5* expression in leaves and roots of Lj11 treated with 60 mM NaHCO_3_ and 5 mM H_2_O_2_ stress. The values of 0 h (control) are set as 1. Values are mean ± SD; n = 3. Statistical analyses were performed using Student’s t test: **p* < 0.05, ***p* < 0.01.


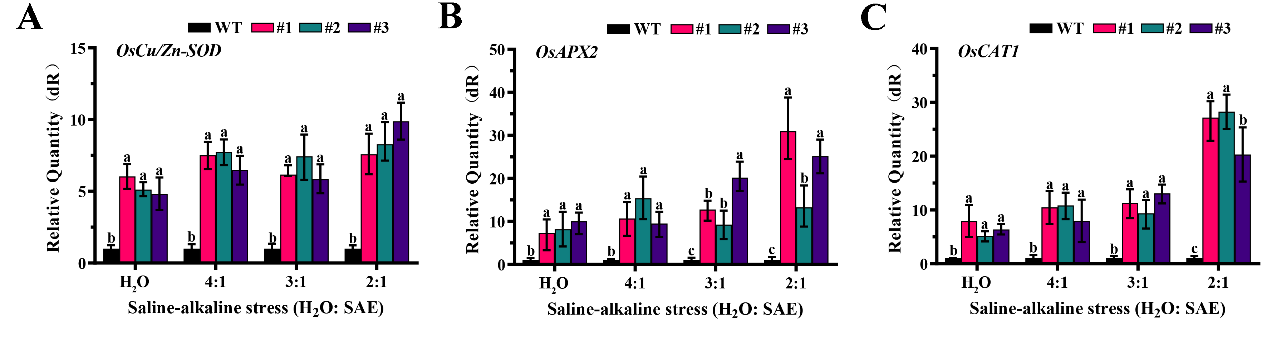


**Figure S7. Expression of *OsCu*/*Zn-SOD*, *OsAPX2* and *OsCAT1* in *OsPK5* overexpression rice under soda saline-alkaline stress.**

7-day-old *OsPK5* overexpression lines and Lj11 seedlings were treated with different ratios of SAE (H_2_O:SAE=4:1, 3:1, and 2:1, water as a control) for 7 days. The expression levels of *OsCu/Zn-SOD*, *OsAPX2* and *OsCAT1* were detected by qRT–PCR. The expression level of Lj11 was set to 1, and the *Os18sRNA* gene was used as an internal reference control. Values are the mean ± standard deviation of three replicates. Statistical differences are labeled with different letters using Duncan test (*p* < 0.05, one-way ANOVA).
